# Editorial: Frontiers' research topic “advances in esophageal cancer surgery with neoadjuvant therapies”

**DOI:** 10.3389/fsurg.2023.1242293

**Published:** 2023-11-15

**Authors:** Qi-Xin Shang, Zhi-Nuan Hong, Ming-Qiang Kang, Long-Qi Chen

**Affiliations:** ^1^ Department of Thoracic Surgery, West China Hospital of Sichuan University, Chengdu, China; ^2^ Department of Thoracic Surgery, Fujian Medical University Union Hospital, Fuzhou, China

**Keywords:** neoadjuvant therapy, esophageal cancer, prognosis factors, risk factors, nomogram model

**Editorial on the Research Topic** Advances in esophageal cancer surgery with neoadjuvant therapies

## Introduction

Esophageal cancer is considered one of the most common cancers globally, characterized by high regional incidence, mortality, and poor prognosis. Locally advanced esophageal cancer accounts for the majority of deaths. Conventional radical resection alone provides insufficient outcomes for these patients. There is now increasing adoption of neoadjuvant therapies followed by surgery. However, neither neoadjuvant chemotherapy nor chemoradiotherapy has yielded promising results for esophageal cancer patients. Thus, there remains an urgent need to identify optimized treatment regimens, multimodality therapies, and immunotherapy combinations to augment the effects of neoadjuvant treatment. Therefore, the topic titled “Advances in Esophageal Cancer Surgery with Neoadjuvant Therapies” was proposed and has collated 12 contributions from experts dedicating in exploring the potential predictive models with clinical significance or prognostic predictors in terms of esophageal cancer patients received the neoadjuvant therapy.

## Development of individualized treatment for patients with locally advanced esophageal cancer

Individualized treatment requires examination methods and tailored therapeutic schemes. Jin et al. found that for esophageal squamous cell carcinoma patients, preoperative radiotherapy (RT) improved overall survival of cT3-4N0M0 patients but not cT1-2N0M0 patients (Jin et al.). Though immunotherapy combined with chemotherapy is proven effective in advanced esophageal cancer, the optimal regimen remains unclear. Zhang et al. found that Sintilimab combined with paclitaxel liposome and carboplatin had a relatively high partial response rate (22.2%) and safety profile, warranting further study (Zhang et al.). Li et al. integrated 15 trials on immunotherapy in neoadjuvant esophageal cancer. R0 resection rates ranged from 80.5% to 100.0%. When neoadjuvant immunotherapy was combined with chemotherapy, pathological complete response ranged from 16.7% to 50.0% and major pathological response ranged from 41.7% to 72.2% (Li et al.). Radiomics involves extracting quantitative imaging features and analyzing related clinical data. Though playing an active role in cancer research, it has little impact on predicting and managing esophageal cancer patients undergoing neoadjuvant therapy. Guo et al. reviewed radiomics' application in this setting. They found that based on CT, PET/CT and MRI radiomics models, pathological complete response after neoadjuvant therapy can be well predicted (Guo et al.). Combining radiomics features with biomarkers like CD44 and SHH further improves accuracy. Radiomics can also perfectly predict recurrence and survival after neoadjuvant therapy.

## Decision of identifying prognostic predictors

Several studies focused on identifying prognostic factors for esophageal cancer patients after neoadjuvant therapy. Reported prognostic predictors include gender, the modified Ryan pathological grading, regional lymph node recurrence, cell senescence-related gene expression signature, and systemic inflammatory markers.

Wang et al.’s study demonstrated that males with locally advanced esophageal cancer had significantly decreased cancer-specific survival after neoadjuvant chemoradiation (*p* < 0.05). Zhang et al. found that the modified Ryan score score was significantly correlated with smoking history, lymphovascular invasion (LVI) and/or peripheral nerve invasion (PNI), however, it's not confirmed as the independent prognostic factors (Wang et al.).

Dai et al. observed that regional lymph node recurrence within 1 year after surgery was the main factor for failure and inferior survival. Irrespective of neoadjuvant therapy, patients with 1-year lymph node recurrence had significantly decreased survival (HR = 11.331, 95% CI 6.870–16.688, *P* < 0.001) with upper thoracic location and N2-3 stage as independent risk factors (Zhang et al.).

Zhang et al. demonstrated that senescence-related genes play a critical role in immune checkpoint regulation. They established that an esophageal cancer senescence-related gene expression signature was negatively correlated with survival (HR = 1.83, 95% CI 1.28–2.59, *p* = 0.004) (Dai et al.).

Han et al.’s meta-analysis indicated that high systemic inflammation levels (SII > 921.80) and early clinical stage were significantly associated with pathological complete response after neoadjuvant induction chemotherapy (OR = 5.32, 95% CI 3.12–9.07, *p* < 0.001), suggesting the feasibility of SII as a predictive tool (Zheng et al.).

## Predictive model construction

Nomograms combine risk factors with predictive factors to assess individual risk and are widely used to aid clinical decision-making. Among the 12 studies, nomogram construction mainly predicted outcomes after neoadjuvant treatment.

For predicting complications after neoadjuvant therapy, Chen et al. established a nomogram to evaluate preoperative anastomotic leakage risk in esophageal cancer patients (Chen et al.). They found that aortic calcification, heart disease, obesity and low FEV1 conferred higher risk. The nomogram's AUC was 0.67, better predicting postoperative leakage. Fang et al. identified preoperative Alb ≤ 41.2 g/L, LA diameter >32.9 mm, Hb > 149 g/L and EF > 67.61% as post-esophagectomy atrial fibrillation (POAF) risk factors. The nomogram (AUC = 0.77) assessed POAF risk to guide individualized treatment (Fang et al.).

For predicting prognosis after neoadjuvant therapy, 7 studies established nomogram models. Wang et al. identified sex, T stage, N stage and M stage as independent cancer-specific survival factors in locally advanced esophageal cancer patients after neoadjuvant chemoradiation (Wang et al.). The nomogram predicted 3-, 5- and 7-year survival (AUC 0.612–0.638). Zhang et al.’s nomogram based on the modified Ryan score had a C-index of 0.702. Similarly, Yang and He determined independent prognostic factors to construct a nomogram for 3-year overall survival (AUC 0.624) in esophageal cancer patients after neo-chemotherapy (Yang and He.). For CSRS and SII studies, the 5-year AUCs of nomograms predicting patient outcomes were 0.946 and 0.62, respectively, both well predicting prognosis and pathological complete response after neoadjuvant therapy.

## Summary

Esophageal cancer has a high morbidity and mortality rate in East Asia, and more than half of the new cases of esophageal cancer all over the world are diagnosed in China. Most patients have suffered from locally advanced disease at the time of diagnosis. At present, surgery is still the basis for the treatment of esophageal cancer. For the treatment of resectable locally advanced esophageal cancer, neoadjuvant therapy combined with surgery may well reduce the volume of tumor and lymph nodes, increase the rate of R0 resection, and prolong survival. With the release of the results of the CROSS trial and the NEOCRTEC5010 trial, preoperative NCRT is the standard treatment for patients with resectable locally advanced esophageal cancer currently. Furthermore, with the FDA's approval of pembrolizumab for the treatment of patients with advanced esophageal cancer in 2019, the era of immunotherapy in esophageal cancer treatment began. However, not all patients with EC will benefit from the neoadjuvant therapy.

How to accurately identify and predict the treatment effect and prognosis of patients who have received neoadjuvant therapy, find out the predictive factors that affect pCR and prognosis, and build a risk prediction model, so as to achieve the goal of individualized treatment. Undoubtedly, in this topic, we have collected a number of studies related to the prognosis of neoadjuvant therapy and pCR results, as well as a review of the current advanced neoadjuvant therapeutic schemes, including immunotherapy. Further, studies are needed in terms of the fields of Esophageal Cancer Surgery with Neoadjuvant Therapies Research.

